# Risk Assessment and Management of COVID-19 Among Travelers Arriving at Designated U.S. Airports, January 17–September 13, 2020

**DOI:** 10.15585/mmwr.mm6945a4

**Published:** 2020-11-13

**Authors:** Philip Dollard, Isabel Griffin, Andre Berro, Nicole J. Cohen, Kimberly Singler, Yoni Haber, Chris de la Motte Hurst, Amber Stolp, Sukhshant Atti, Leslie Hausman, Caitlin E. Shockey, Shahrokh Roohi, Clive M. Brown, Lisa D. Rotz, Martin S. Cetron, CDC COVID-19 Port of Entry Team, Francisco Alvarado-Ramy

**Affiliations:** ^1^CDC COVID-19 Response Team; ^2^Division of Global Migration and Quarantine, National Center for Emerging and Zoonotic Infectious Diseases, CDC; ^3^Oak Ridge Institute for Science and Education, Oak Ridge, Tennessee; ^4^Kapili Services, LLC, Orlando, Florida.

In January 2020, with support from the U.S. Department of Homeland Security (DHS), CDC instituted an enhanced entry risk assessment and management (screening) program for air passengers arriving from certain countries with widespread, sustained transmission of SARS-CoV-2, the virus that causes coronavirus disease 2019 (COVID-19). The objectives of the screening program were to reduce the importation of COVID-19 cases into the United States and slow subsequent spread within states. Screening aimed to identify travelers with COVID-19–like illness or who had a known exposure to a person with COVID-19 and separate them from others. Screening also aimed to inform all screened travelers about self-monitoring and other recommendations to prevent disease spread and obtain their contact information to share with public health authorities in destination states. CDC delegated postarrival management of crew members to airline occupational health programs by issuing joint guidance with the Federal Aviation Administration.* During January 17–September 13, 2020, a total of 766,044 travelers were screened, 298 (0.04%) of whom met criteria for public health assessment; 35 (0.005%) were tested for SARS-CoV-2, and nine (0.001%) had a positive test result. CDC shared contact information with states for approximately 68% of screened travelers because of data collection challenges and some states’ opting out of receiving data. The low case detection rate of this resource-intensive program highlighted the need for fundamental change in the U.S. border health strategy. Because SARS-CoV-2 infection and transmission can occur in the absence of symptoms and because the symptoms of COVID-19 are nonspecific, symptom-based screening programs are ineffective for case detection. Since the screening program ended on September 14, 2020, efforts to reduce COVID-19 importation have focused on enhancing communications with travelers to promote recommended preventive measures, reinforcing mechanisms to refer overtly ill travelers to CDC, and enhancing public health response capacity at ports of entry. More efficient collection of contact information for international air passengers before arrival and real-time transfer of data to U.S. health departments would facilitate timely postarrival public health management, including contact tracing, when indicated. Incorporating health attestations, predeparture and postarrival testing, and a period of limited movement after higher-risk travel, might reduce risk for transmission during travel and translocation of SARS-CoV-2 between geographic areas and help guide more individualized postarrival recommendations.

On January 17, 2020, entry screening of air passengers arriving from Wuhan, Hubei Province, China, the epicenter of the COVID-19 outbreak at the time, began at three U.S. airports (Los Angeles International Airport, California; San Francisco International Airport, California; and John F. Kennedy International Airport, New York City, New York) receiving the highest volume of passengers arriving from Wuhan Tianhe International Airport (Table 1) (Figure). Beginning February 3, entry screening expanded to all passengers arriving from mainland China after the issuance of a presidential proclamation^†^ restricting entry to U.S. citizens, lawful permanent residents, and other excepted persons. These travelers were routed to one of 11 designated airports. On March 2, travelers from Iran were added.^§^ As Europe became a new epicenter of COVID-19, travelers from 26 countries in the European Schengen Area^¶^ (effective March 14), the United Kingdom, and Ireland** (effective for both March 17) were added, and the number of airports to which passengers were routed expanded to 13. When travelers from Brazil^††^ were added on May 28, screening expanded to 15 designated airports.

**TABLE 1:** Airports participating in COVID-19 entry screening operations and volume of passengers screened, by date of initiation of screening (N = 15) — United States, January 17–September 13, 2020

Date screening began	Screening airport	City, State	IATA code	No. of passengers screened (%)
Jan 17, 2020	John F. Kennedy International	New York City, New York	JFK	146,127 (19.1)
	Los Angeles International	Los Angeles, California	LAX	79,486 (10.4)
	San Francisco International	San Francisco, California	SFO	45,237 (5.9)
Jan 21, 2020	O’Hare International	Chicago, Illinois	ORD	86,412 (11.3)
	Hartsfield-Jackson Atlanta International	Atlanta, Georgia	ATL	78,893 (10.3)
Feb 3, 2020	Newark Liberty International	Newark, New Jersey	EWR	79,507 (10.4)
	Washington Dulles International	Dulles, Virginia	IAD	66,107 (8.6)
	Dallas-Fort Worth International	Dallas, Texas	DFW	45,289 (5.9)
	Detroit Metropolitan	Detroit, Michigan	DTW	24,739 (3.2)
	Seattle-Tacoma International	Seattle, Washington	SEA	11,781 (1.5)
	Daniel K. Inouye International	Honolulu, Hawaii	HNL	1,052 (0.1)
Mar 14, 2020	Miami International	Miami, Florida	MIA	40,871 (5.3)
	Boston Logan International	Boston, Massachusetts	BOS	38,937 (5.1)
	George Bush Intercontinental	Houston, Texas	IAH	15,024 (2.0)
	Fort Lauderdale-Hollywood International	Fort Lauderdale, Florida	FLL	6,582 (0.9)
**Total**	**—**	**—**	**—**	**766,044 (100)**

**Abbreviations:** COVID-19 = coronavirus disease 2019; IATA = International Air Transport Association.

**FIGURE:**
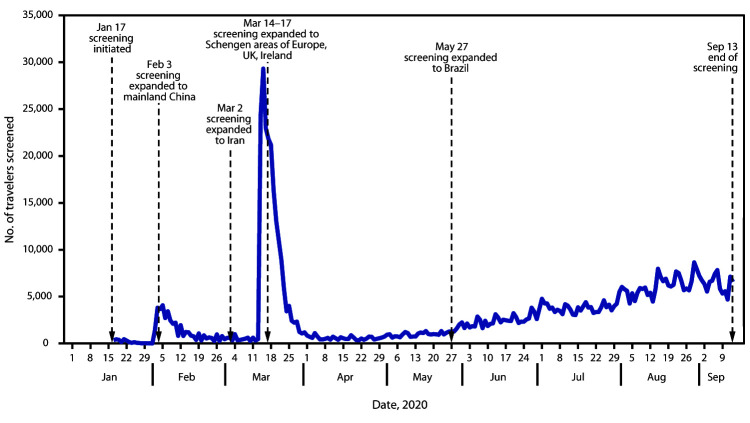
Number of travelers screened for COVID-19 and changes in screening program — 15 designated U.S. airports, January 17–September 13, 2020 **Abbreviations:** COVID-19 = coronavirus disease 2019; UK = United Kingdom.

Screening consisted of three steps. First, U.S. Customs and Border Protection officers identified and referred travelers for screening if they had been in one of the specified countries during the previous 14 days. Next, initial screening was conducted, which included observation for signs of illness, a temperature check using a noncontact infrared thermometer (fever defined as temperature ≥100.4°F [38°C]), administration of a questionnaire about signs and symptoms (fever, cough, and difficulty breathing) in the preceding 24 hours or exposure to a person with COVID-19 in the preceding 14 days, and collection of travelers’ U.S. contact information. The third step included referral of ill travelers and those disclosing an exposure for additional public health assessment by an on-site medical officer; if indicated, travelers were sent to a local health care facility for medical evaluation. The threshold for sending symptomatic travelers for public health assessment and deciding which among those would be sent for medical evaluation varied during the evaluation period, reflecting evolution of CDC’s definition for “person under investigation”^§§^ and operational considerations (e.g., testing capacity). Until March 20, travelers from Hubei Province were quarantined for 14 days upon arrival under federal or state authority.

All screened travelers received a Travel Health Alert Notice, an information card that advised them to stay home (or in a comparable setting, such as a hotel room) for 14 days after arrival and provided messaging on self-monitoring for COVID-19 symptoms and actions to take if symptoms develop. Traveler contact information was transmitted securely to state health departments via CDC’s Epidemic Information Exchange (Epi-X). In addition to covering all costs for CDC personnel and contractors conducting screening, CDC transferred about $57 million to DHS to support the screening operation and incurred additional costs for equipment, travel, and housing of quarantined travelers. At the program’s peak volume on March 20, designated airports were staffed with approximately 750 screeners, plus other supporting personnel.

During January 17–September 13, 2020, 766,044 travelers were screened (Table 1), 298 (0.04%) of whom met CDC criteria for referral. Travelers were referred because they had either been in Hubei Province (16, 5.4%), reported contact with a person with COVID-19 (four, 1.3%), or had signs or symptoms that triggered a public health assessment (278, 93.3%). Among the 278 persons who had COVID-19–like symptoms, the most common signs or symptoms triggering assessment were cough (73%), self-reported fever (41%), measured fever (17%), and difficulty breathing (13%) (Table 2). Forty (14%) of these travelers were medically evaluated at a local health care facility, and 35 (13%) were tested for SARS-CoV-2 using reverse transcription–polymerase chain reaction (RT-PCR); nine of the 35 tests returned positive results, representing 0.001% (one per 85,000) of all travelers screened. Fourteen additional travelers with laboratory-confirmed COVID-19 were identified through other mechanisms rather than as a direct result of entry screening: six via established processes with airlines and airport partners to detect ill travelers and notify CDC and eight through notifications about travelers who had received a positive test result in the United States or another country before travel.

**TABLE 2:** Characteristics of symptomatic travelers screened for COVID-19 at U.S. airports who were referred for on-site public health assessment, tested for SARS-CoV-2, and who received a diagnosis of laboratory-confirmed SARS-CoV-2 infection — 15 U.S. airports, January 17–September 13, 2020

Sign/Symptom	No. (%) among symptomatic travelers		Laboratory-confirmed COVID-19, no. (% of symptomatic travelers), [% of tested travelers] (n = 9)
	Referred for public health assessment (n = 278)	Received SARS-CoV-2 testing (n = 35)	
Cough	202 (72.7)	28 (10.1)	7 (2.5), [20.0]
Self-reported fever	113 (40.6)	27 (9.7)	8 (2.9), [22.9]
Measured fever (temperature ≥100.4°F [38°C])	48 (17.3)	15 (5.4)	5 (1.8), [14.3]
Difficulty breathing	36 (12.9)	17 (6.1)	2 (0.7), [5.7]
Measured fever plus difficulty breathing	2 (0.7)	2 (0.7)	0 (0), [0]
Measured fever plus cough	10 (3.6)	7 (2.5)	3 (1.1), [8.6]

**Abbreviation:** COVID-19 = coronavirus disease 2019.

CDC relied initially on existing federal traveler databases to obtain passenger contact information to share with states, but missing or inaccurate data prompted adding manual data collection to the screening process. Manual data collection resulted in 98.1% complete records (i.e., records contained both phone number and physical address). CDC sent state health departments contact information for approximately 68% of screened travelers. CDC did not send records processed 12 days after travelers’ arrival, with insufficient contact data, or belonging to six states that opted out of receiving travelers’ data because of competing response priorities. Analysis of traveler data submitted electronically by airlines during September 14–24, after discontinuation of manual data collection, and supplemented by previously untapped federal databases, showed that 22% of traveler contact information records had phone number and physical address.

## Discussion

These findings demonstrate that temperature and symptom screening at airports detected few COVID-19 cases and required considerable resources. The observed yield was approximately one identified case per 85,000 travelers screened. Reasons for the low yield were likely multifactorial and might have included an overall low COVID-19 prevalence in travelers; the relatively long incubation period; an illness presentation with a wide range of severity, afebrile cases, and nonspecific symptoms common to other infections; asymptomatic infections; and travelers who might deny symptoms or take steps to avoid detection of illness (e.g., through use of antipyretic or cough suppressant medications) (*1*).

SARS-CoV-2 presents a formidable control challenge because asymptomatic (i.e., never symptomatic) and presymptomatic (i.e., contagious infections before symptom onset) infections can result in substantial transmission, which was unknown early in the pandemic (*2*,*3*). The proxy for infectiousness, viral shedding in the upper respiratory tract, is greatest early in the course of infection, before prominent symptoms are apparent, suggesting peak infectiousness at or before symptom onset (*3*).

These findings are consistent with mathematical models examining the effectiveness of airport screening for COVID-19, which suggest that most infected travelers would be undetected by symptom-based screening at airports (*4*,*5*). Nonetheless, reductions in travel (e.g., associated with issuance of travel health notices to avoid nonessential travel and some entry restrictions) and airport-based activities might have lessened the incidence of COVID-19 in the United States early in the pandemic by discouraging symptomatic persons from traveling, limiting entry of potentially infected travelers, and promoting actions to prevent transmission from infected travelers, including a recommendation to stay home for 14 days after arrival (*6*–*8*).

Challenges associated with providing complete and accurate traveler contact information to health departments, the high volume of travelers to some locations, and competing health department priorities when jurisdictions were confronting outbreaks, precluded efforts to contact most travelers after arrival to oversee self-monitoring as recommended at the time (*9*). Manual data collection of traveler contact information on arrival is resource-intensive and poses a risk to travelers who might have to wait in crowded, enclosed spaces while the information is collected. CDC is working with government and industry partners to develop a framework to collect reliable contact information electronically for airline passengers before arrival in the United States and enable secure, real-time data transfer for any public health follow-up, including air travel-related contact tracing, when indicated.

The findings in this report are subject to at least three limitations. First, not all symptomatic travelers were referred for public health assessment because many COVID-19 symptoms are nonspecific and available data (for travelers who were not referred) are insufficient to determine the proportion who might have had some symptoms. Second, most travelers referred for public health assessment were not sent to a local health care facility or tested for SARS-CoV-2. Both could have been sources of selection bias toward underestimation of the number of cases in screened travelers. Third, screening was limited to travelers from certain countries, and current surveillance systems lack information to match COVID-19 cases reported by states to known international travelers. Therefore, this report is unable to provide definitive assessment of the outcomes of screened travelers who were not referred for medical evaluation or to compare outcomes for screened travelers with those arriving from countries not targeted for screening.

The hallmark of effective public health programs is reassessment of methods used for public health practice based on available evidence. Therefore, CDC recommended a shift from resource-intensive, low-yield, symptom-based screening of air travelers to an approach that better fits the current stage of the pandemic, and on September 14, 2020, the screening program was discontinued. Protecting travelers and destination communities during the pandemic will require continued emphasis on implementation of health precautions before, during, and after travel, and communicating these recommendations to travelers and the airline industry.^¶¶^^,^***^,^^†††^^,^^§§§^ After the removal of requirements for enhanced entry screening operations, CDC continued to invest in strengthening illness detection and response under CDC’s regulatory authorities,^¶¶¶^ by training of partners at ports of entry, as well as overall public health response capacity at 20 CDC quarantine station locations. CDC, along with U.S. government partners, also issued recommendations for airlines, airports, and travelers****^,^^††††^ to prevent COVID-19 transmission associated with air travel. All travelers should follow CDC recommendations for mask use,^§§§§^ hand hygiene, self-monitoring for symptoms, and social distancing during travel and after arrival to the United States. Travellers with higher exposure risk should take additional precautions, including postarrival testing, avoiding contact with persons at higher risk for severe disease, and staying home as recommended or required by jurisdictional public health authorities. Predeparture testing of travelers, ideally with specimen collection within 72 hours before departure, might reduce the risk for SARS-CoV-2 transmission during travel. Postarrival testing could allow for shortening of posttravel self-quarantine periods that protect against travel-associated imported (translocated) infections.^¶¶¶¶^ Finally, progress in understanding immunity biomarkers and duration of protection, in developing one or more vaccines, and in testing hold promise for refining risk stratification and optimizing management of travelers to reduce COVID-19 transmission and translocation related to commercial air travel.

**Summary**

**What is already known about this topic?**

As an early effort to prevent importation of SARS-CoV-2, CDC established entry screening at designated airports for passengers from certain countries.

**What is added by this report?**

Passenger entry screening was resource-intensive with low yield of laboratory-diagnosed COVID-19 cases (one case per 85,000 travelers screened). Contact information was missing for a substantial proportion of screened travelers in the absence of manual data collection.

**What are the implications for public health practice?**

Symptom-based screening programs are ineffective because of the nonspecific clinical presentation of COVID-19 and asymptomatic cases. Reducing COVID-19 importation has transitioned to enhancing communication with travelers to promote recommended preventive measures, strengthening response capacity at ports of entry, and encouraging predeparture and postarrival testing. Collection of contact information from international air passengers before arrival would facilitate timely postarrival management when indicated.

## Footnotes

* https://www.faa.gov/other_visit/aviation_industry/airline_operators/airline_safety/safo/all_safos/media/2020/SAFO20009.pdf.

†
https://www.whitehouse.gov/presidential-actions/proclamation-suspension-entry-immigrants-nonimmigrants-persons-pose-risk-transmitting-2019-novel-coronavirus/ .

§
https://www.whitehouse.gov/presidential-actions/proclamation-suspension-entry-immigrants-nonimmigrants-certain-additional-persons-pose-risk-transmitting-coronavirus/ .

¶
https://www.whitehouse.gov/presidential-actions/proclamation-suspension-entry-immigrants-nonimmigrants-certain-additional-persons-pose-risk-transmitting-2019-novel-coronavirus/ .

** https://www.whitehouse.gov/presidential-actions/proclamation-suspension-entry-immigrants-nonimmigrants-certain-additional-persons-pose-risk-transmitting-coronavirus-2/.

††
https://www.whitehouse.gov/presidential-actions/proclamation-suspension-entry-immigrants-nonimmigrants-certain-additional-persons-pose-risk-transmitting-novel-coronavirus/ .

§§ https://www.emergency.cdc.gov/han/2020.asp.

¶¶
https://www.cdc.gov/coronavirus/2019-ncov/travelers/when-to-delay-travel.html .

*** https://www.cdc.gov/coronavirus/2019-ncov/travelers/travel-during-covid19.html.

†††
https://www.cdc.gov/coronavirus/2019-ncov/travelers/after-travel-precautions.html .

§§§
https://www.cdc.gov/quarantine/travel-restrictions.html .

¶¶¶
https://www.cdc.gov/quarantine/specificlawsregulations.html .

****
https://www.cdc.gov/coronavirus/2019-ncov/travelers/airline-toolkit.html .

††††
https://www.transportation.gov/sites/dot.gov/files/2020-07/Runway_to_Recovery_07022020.pdf .

§§§§
https://www.cdc.gov/quarantine/masks/mask-travel-guidance.html .

¶¶¶¶ https://www.medrxiv.org/content/10.1101/2020.07.24.20161281v2.full.pdf.
